# Molecular Genotyping of *Toxoplasma gondii* in Sheep Aborted Fetuses Reveals Predominance of Type I Infection in Southwest of Iran

**DOI:** 10.18502/ijpa.v15i3.4202

**Published:** 2020

**Authors:** Nasir AREFKHAH, Bahador SARKARI, Qasem ASGARI, Abdolali MOSHFE, Mohammad Hasan KHALAFI, Iraj MOHAMMADPOUR

**Affiliations:** 1. Department of Parasitology and Mycology, School of Medicine, Shiraz University of Medical Sciences, Shiraz, Iran; 2. Cellular and Molecular Research Center, Yasuj University of Medical Sciences, Yasuj, Iran; 3. Basic Sciences in Infectious Diseases Research Center, Shiraz University of Medical Sciences, Shiraz, Iran; 4. Veterinary Organization of Kohgiluyeh and Boyer-Ahmad Province, Yasuj, Iran

**Keywords:** *Toxoplasma gondii*, Ovine aborted fetuses, Sequence analysis, Iran

## Abstract

**Background:**

We aimed to detect *Toxoplasma gondii* in ovine aborted fetuses and evaluate its genetic variations in the southwest of Iran.

**Methods:**

This cross-sectional study was performed on 100 aborted ovine fetuses collected from the different region of Kohgiluyeh and Boyer-Ahmad Province, Iran, in lambing season during 2017 and 2018. DNA was extracted from the brain samples of all of the aborted fetuses and PCR amplified, targeting a 529 bp repetitive element gene of *T. gondii*. Moreover, to find out the heterogeneity of the positive samples, PCR-DNA amplification of the two main genetic markers, B1 and GRA6, of *T. gondii* were performed. Nucleotide sequencing and phylogenetic analysis were performed, using the BLAST program and MEGA-X software.

**Results:**

The 529 bp gene of *T. gondii* was detected in 2 out of 100 (2%) of the ovine aborted samples. The sequences analysis of GRA6 and B1 genes revealed that both isolates from the aborted fetuses of sheep belonged to type I of *T. gondii.* Intra-divergence was more seen in GRA6 gene whereas less divergence was observed in B1 gene.

**Conclusion:**

Congenital infection with Type I of *T. gondii* during the neonatal period is associated with abortion in ovine. Evaluation of more aborted samples from broader geographical areas is needed to elucidate the molecular epidemiology and also the genotypes of *T. gondii* associated with abortion.

## Introduction

There are broad ranges of microorganism which may cause abortion in small ruminants as well as human. *Toxoplasma gondii* is one of the most prevalent food-borne zoonotic pathogens with the medical and veterinary importance. *Toxoplasma-*related abortion continues to be an important worldwide challenge not only in the human population but also in sheep and goat breeding industries (1–4).

Previous studies in different areas of the world have shown that *T. gondii* could appear as a life-threatening zoonotic parasite of fetuses of sheep, causing considerable economic losses (1). For example, the costs of congenital human toxoplasmosis in the UK and annual loss for ovine toxoplasmosis in Brazil have been estimated at $1.2–12 and $1.491 million respectively (5, 6). The prevalence rate of toxoplasmosis in aborted fetuses in different areas of the World have been reported to be 23.1% in Spain, 18.1 % in Italy, 17.5% in the USA, and 10.6% in Germany (7–10).

All mammals and bird’s species can serve as intermediate hosts for *T. gondii*; however, sheep is considered as one of the most susceptible animals that are mainly infected through consumption of water or pastures contaminated with oocysts dispersed by cats, and also via the congenital route (4).

*T. gondii* infection in adult sheep is usually subclinical. However, maternal-fetal transmission of toxoplasmosis depends on the stage of gestation infection, has great consequences as *T. gondii* infection during pregnancy may result in mummified, macerated, abortion or stillborn, and other disorders (11).

*Toxoplasma* populations show a clonal genetic structure including of three canonical types (type I, II, and III) and also the non-clonal or “atypical” type. The *T. gondii* clonal type is considered as the main factor in the clinical presentation of toxoplasmosis in mice and previous studies have shown that this may also apply to other intermediate hosts, for instance, ruminants and humans (3, 9, 12). For example, type I is lethal to out-bred mice while type II and III isolates are less virulent. There is growing concern about the high prevalence of toxoplasmosis in sheep and cats in Iran (4, 13, 14). This, in turn, can increase the chance of congenital toxoplasmosis both in humans and animals.

Study of reproductive failure linked to the genotypes of *Toxoplasma* in ovine aborted fetuses has fundamental importance. PCR method, using the 200-300-fold repetitive 529 bp element, is a useful diagnostic target with high sensitivity and specificity for detection of the *T. gondii* infection (15). Moreover, the B1 gene, with the appropriate rate of variability, is considered a suitable target for molecular characterization of *T. gondii*. Likewise, the GRA6 gene, with a very high rate of DNA polymorphism, is considered as one of the most suitable markers for evaluating the genetic diversity of *T. gondii* which clearly differentiate the three different genetic types and also some of the atypical genotypes of the parasite (16, 17). Data regarding to genotyping of *T. gondii* in aborted fetuses is lacking in most areas of Iran.

We aime to assess the genotypes and the genetic variations of *T. gondii* in ovine aborted fetuses in Kohgiluyeh and Boyer-Ahmad Province in the southwest of Iran.

## Materials and Methods

### The study area

**Fig. 1:**
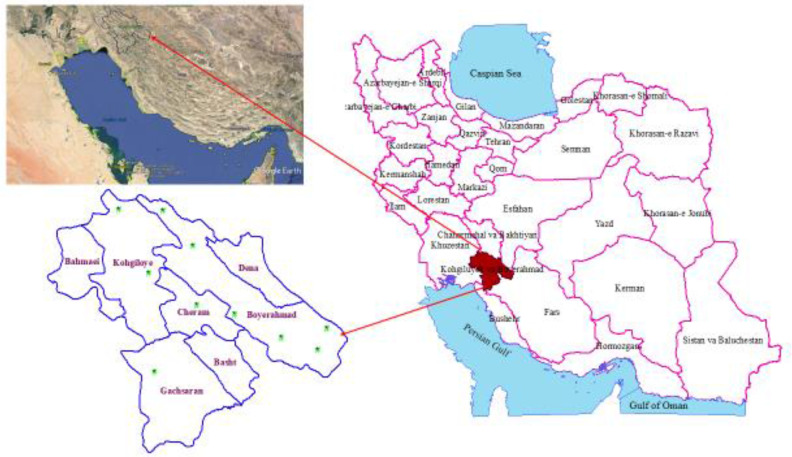
Geographical location of Kohgiluyeh and Boyer-Ahmad province in the southwest of Iran and the studied areas

This cross-sectional study was performed from Apr 2017 to Mar 2018 on aborted fetuses of sheep in Kohgiluyeh and Boyer-Ahmad Province, in the southwest of Iran (Fig. 1). The district is known as an important sheep production where animal husbandry is quite common. The criterion for choosing the area was the high prevalence of abortion and early embryonic death in sheep as reported by the veterinary organization in the Province. The area is an intriguing region for animal husbandry in summer and spring. Moreover, in autumn and winter seasons, some of the livestock breeders migrate to the Dehdasht and Gachsaran regions, in the southern part of the Province with a tropical weather, to find pasture for their livestock.

### Sampling

Samples were collected from the naturally occurring fresh aborted ovine fetuses of sheep from different regions of the Province. Fetuses with dystocia during the delivery were excluded from the study. The sample size was calculated considering the prevalence rate of ovine aborted fetuses, and 95% confidence intervals. During the lambing season (early Apr to late Jun), 100 sheep aborted fetuses in different areas of the Province were collected. From each freshly aborted fetus a part of the brain tissue was removed, stored in 70% ethanol and transferred to the immunoparasitology laboratory at Shiraz University of Medical Sciences (SUMS) where they were stored at − 20 °C until use.

The Ethics Committee of Shiraz University of Medical Sciences approved the current study protocol.

### DNA extraction and PCR

Genomic DNA was extracted from each brain sample of aborted ovine fetuses, using Tissue Genomic DNA Extraction kit (Favorgene Biotech Corp, Taiwan) in accordance with the manufacturer’s instructions. The DNA concentration was determined by NanoDrop. For screening of the samples, the extracted DNA were amplified by PCR, targeting a 529 bp repetitive element gene of *T. gondii,* using ToxoF (5′- CAG GGA GGA AGA CGA AAG TTG - 3′) and ToxoR (5′- CAG ACA CAG TGC ATC TGG ATT- 3′) primers (15). The PCR reactions were performed in an Eppendorf Master cycler Gradient (Eppendorf, Hamburg, Germany) in a 25-μL reaction volume that contained 15 ng of template DNA, 12.5 μL of 2× Taq PCR mix (Amplicon, Odense, Denmark), 1 μL of each primer (10 pmol/ μL), and 9.5 μL of ddH_2_O. Cycling program was initiated at 95 °C for 5 min, followed by 35 cycles at 95 °C for 45s, 60 °C for 45 s, 72 °C for 45s and finished with 72 °C for 5 min. A positive control from *T. gondii* tachyzoites and negative control were included in every experiment. PCR products were visualized by electrophoresis on a 1.5% agarose gel stained with Gel Red nucleic acid gel stain (GelRed®, Biotium, CA, USA), and examined under a BioDoc gel documentation System (UVP, Upland, CA, USA). The expected length of the amplified DNA fragment was about 529 bp. Afterward, PCR assay was carried out on the positive samples using two different target genes; B1gene using B1F (5′- GGAACTGCATCCGTTCATGAG- 3′) and B1R (5′- TCTTTAAAGCGTTCGTGGTC - 3′) primers, and the GRA6 gene, using a pair of PCR primers consisted of GRA6 forward (5′-TTTCCGAGCAGGTGACCT-3′) and GRA6 reverse (5′- TCGCCGAAGAGTTGACATAG-3′) primers (18,19). For the amplification of B1 and GRA6 genes, PCR reaction volumes were the same as described for 529 bp gene and the PCR reaction conditions were consisted of denaturation at 94 °C for 5 min followed by 35 cycles of 94 °C for 35 S, 56 °C for 45 s, 72 °C for 1 min, and a final elongation step of 72 °C for 10 min. After staining with GelRed nucleic acid gel stain, PCR products were electrophoresed on 1.5% agarose gel. The amplicons were visualized under UV illumination. The expected size of the amplified DNA fragments was 194 and 344 bp for B1 and GRA6 gene, respectively.

### DNA sequencing and phylogenetic analysis

Using the same primers as described for the amplification process, the PCR product was sequenced (Pishgam Company, Tehran, Iran). Sequence analysis of B1 and GRA6 genes were performed to determine the *T. gondii* strain. The sequences were analyzed by Basic Local Alignment Search Tool (BLAST), after being edited with BioEdit Program, and compared with those of available relevant sequences in the GenBank. The phylogenetic relationship among genotypes were estimated using Maximum-likelihood analysis, based on the Kimura 2-parameter model. Mega-X software was also used to construct the phylogenetic trees, using a strain of a *Giardia intestinalis* as the out-group.

## Results

**Fig. 2:**
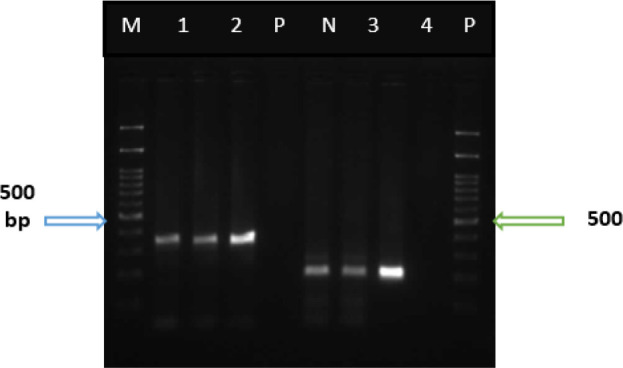
PCR products of isolated *T. gondii* from the brain of aborted fetuses of sheep. M, 100 bp molecular weight marker; P, positive control; N, negative control; lanes 1 and 2, PCR product of GRA6 gene, using DNA isolated from the brain tissue of the aborted fetuses; lanes 3 and 4, PCR product of B1 gene, using DNA isolated from the brain tissue of aborted fetuses

The PCR amplification of the 529 bp gene of *T. gondii* from all of the 100 brain tissue samples of the aborted fetuses yielded a fragment of about 529 bp in 2 (2%) cases (Fig. 2). Moreover, the B1 and GRA6 genes of the positive cases were PCR-amplified which yielded a 194 bp and a 344 bp bands for B1and GRA6 gene, respectively. The PCR products of B1 and GRA6 genes were sequenced and the results were analyzed by BLAST program. Sequencing and phylogenetic analysis of GRA6 and B1 markers revealed that both of the isolates have the most similarity with type I of *T. gondii*. Sequences of genes obtained from the present study were deposited in GenBank, under accession numbers MH899199, and MH899200 for GRA6 and MH899197 and MH899198 for B1 genes.

The sequence of the *T. gondii* GRA6 gene from this study (MH899199) showed 96% homologies to KX231336 and LC414527 (Iran), 96% to MG587986 and MG587983 (Italy), 95% to MG587966 (Italy), and KX781158 (China).

The sequence of the other GRA6 gene (MH899200) of the current study showed 99% homologies to KX231335 (Iran), 98% to LC414527 (Iran), 98% to MG587986 (Italy), and 98% to MG587983 (Italy).

The percentage of identities between the isolates from this study and the *T. gondii* KX270373 (Mexico), *T. gondii* KU877882 (Bangladesh) and *T. gondii* LC057651 (Iran) for the B1 gene were 99 %, 99 %, and 98%, respectively.

**Fig. 3:**
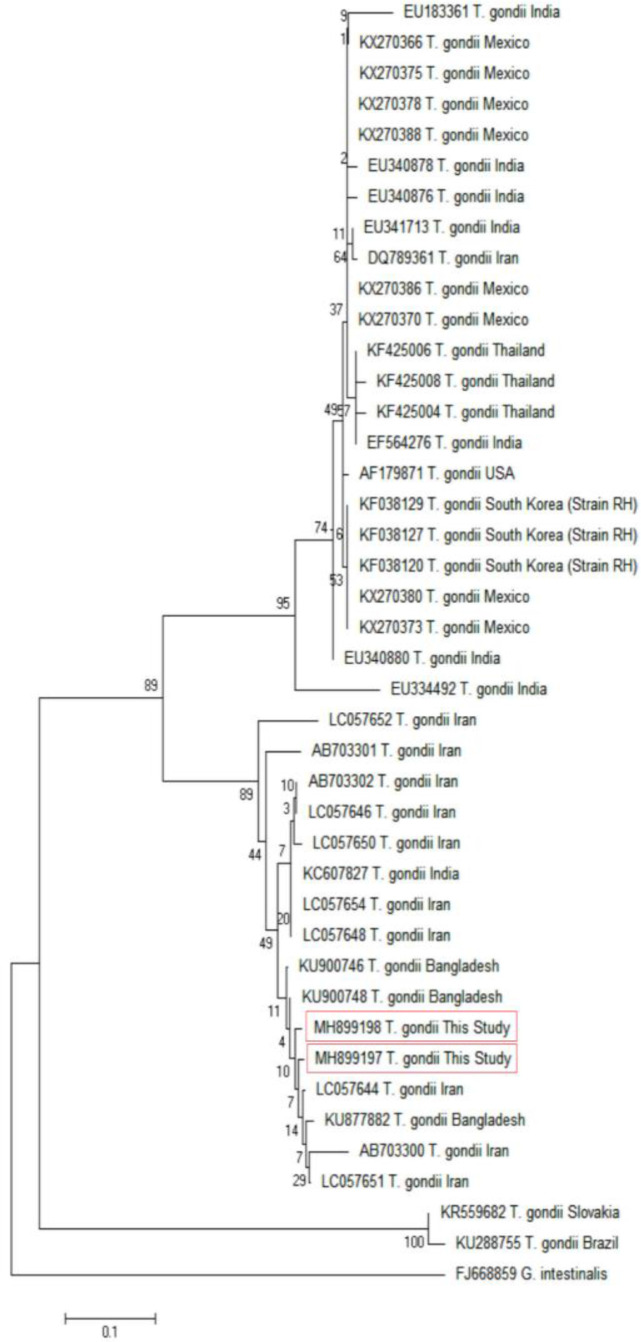
Phylogenetic relationship among various *Toxoplasma gondii* isolates to each other as inferred by Neighbor-Joining tree based on B1 gene. Numbers on branches are percentage bootstrap values of 1,000 replicates. The evolutionary distances between sequences were computed using the Maximum Composite Likelihood method. The scale bar indicates an evolutionary distance of 0.1 nucleotides per position in the sequence. The reference sequences accession numbers are inserted. Evolutionary analyses were conducted in MEGAX

Considering the GRA6 sequences, the phylogenetic analysis of the two identified *T. gondii* isolates were taxonomically grouped into one clade. Within the clade, the published isolates from Iran, Italy, China, and France (strain RH) were positioned (Fig. 3).

**Fig. 4:**
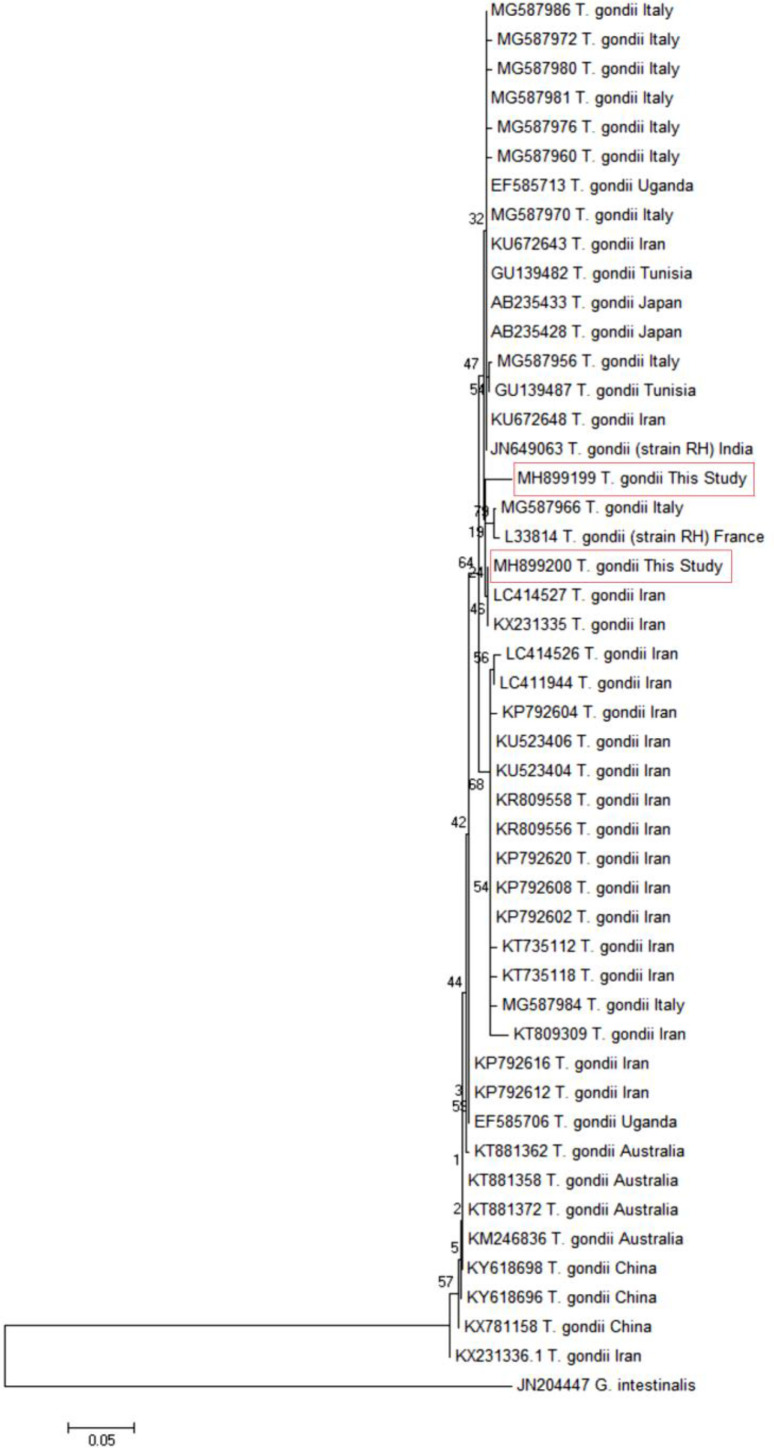
Phylogenetic relationship among various *Toxoplasma gondii* isolates to each other as inferred by Neighbor-Joining tree based on GRA6 gene. Numbers on branches are percentage bootstrap values of 1,000 replicates. The evolutionary distances between sequences were computed using the Maximum Composite Likelihood method. The scale bar indicates an evolutionary distance of 0.05 nucleotides per position in the sequence. The reference sequences accession numbers are inserted. Evolutionary analyses were conducted in MEGAX

Considering the B1 sequences, the phylogenetic analysis of the two identified isolates were taxonomically grouped into one clade. Within the clade, the published isolates from Iran, Bangladesh, India and South Korea (strain RH) were positioned (Fig. 4).

Intra-divergence was more in GRA6 gene especially in isolate with accession number MH899199, while less divergence was observed in the B1 gene.

## Discussion

The economic impact of ovine toxoplasmosis is not yet clear in Iran although the role of *T. gondii* in the abortion of sheep fetuses has been documented in few studies (20, 21).

There is no information on the prevalence of *Toxoplasma* and its abortion rate in sheep in Kohgiluyeh and Boyer-Aahmad region. Therefore, it is necessary to assess the prevalence rate of this parasite and the possible factors that contribute to the reduction of the number of abortion cases with *Toxoplasma* in sheep in such areas.

The current study, for the first time, was performed in Kohgiluyeh and Boyer-Ahmad Province in the southwest of Iran, to detect the DNA of *T. gondii* in aborted fetuses and to evaluate the genetic diversities of B1 and GRA6 target genes of the organism in naturally occurring sheep miscarriages. A prevalence of 2% for *T. gondii* infection was found in ovine aborted brain specimens. This means that about 2% of the aborted fetuses are linked to the infection with *Toxoplasma* during the pregnancy. The prevalence rate of toxoplasmosis in our study is lower than previous reports of aborted fetuses from other regions of Iran such as in Qazvin Province, central Iran (22), and Khorasan Razavi Province in Northeastern Iran (23–25). Moreover, the rate of *T. gondii* infection in aborted fetuses in our study is lower than those reported from Jordan, Brazil, Italy, or from Spain (26–29).

The low prevalence of *Toxoplasma* in aborted samples in our study might be due to many factors including the climatic condition of the studied region, farm size, and host resistance to *Toxoplasma* in some sheep, the severity of *T. gondii* infections in examined tissues, and also the applied PCR assay. Besides, livestock management and the low density of cats in the farm of the region, and less contact between sheep and infected resources such as drinking water and pasture contaminated by cat feces may be accounted for the lower density of *T. gondii* in the aborted samples.

Farmers and ranchers in Kohgiluyeh and Boyer Ahmad usually keep dogs. This leads to a decrease in the number of cats in the region, which in turn reduces the environmental contamination by the oocysts of the parasite defecated by cats. Moreover, mountainous conditions and high altitudes in Yasuj Township (capital of the Province) and low humidity accompanied with high temperatures of soil in Gachsaran and Dehdasht Townships (southern parts of the Province) decreases the viability of *Toxoplasma* oocysts in such environments.

*T. gondii* genetically comprises three main clonal genotypes, including types I, II, and III. Type II and III as the predominance types from adult sheep reported in Iran (17).

In the current study, sequences of the isolates had the highest similarity with type I clonal lineage of *T. gondii*. This finding is in good agreement with similar studies conducted on ovine aborted fetuses in Qazvin and Khorasan Razavi Province, as well as on adult sheep from Jahrom district in Fars province in Iran (22, 24, 30). However, this finding is not consistent with studies conducted in United Kingdom, Italy, (31), Denmark (32), or Ireland (33) that reported type II of *T. gondii* as the most prevalent clonal lineage in aborted fetuses of sheep. This indicates that different genotypes of *T. gondii* parasite is circulating in different areas of the world which are responsible for the abortion in sheep.

Different factors may affect the variations of genotype in different areas of the world which are; differences in geographical location, the degree of parasite genetic diversity for a particular region, sensitivity and also polymorphism of the employed genetic markers, and the number of applied polymorphism locus.

In this study, phylogenetic analysis of the GRA6 and B1 genes indicated that both of our isolates were closely related to RH strains of type I of *T. gondii*. Moreover, the findings revealed that the GRA6 marker appropriately shows the similarity and variations of our isolates with isolates from the West and East parts of Iran. The findings also revealed that the GRA6 gene, in comparison with the B1 gene, shows more divergent features of the isolates. This finding is consistent with the study performed by Fazaeli et al., that demonstrated a high degree of genetic variability for GRA6 (18).

## Conclusion

Type I genotype of *T. gondii* might be considered as a possible cause of neonatal losses in case of naturally occurring miscarriages in the sheep. Further studies are needed to obtain more solid information regarding the molecular epidemiology and genotypes of *T. gondii* by collecting more ovine aborted fetuses from broader geographical areas as well as using more polymorphic markers. It is also necessary to provide more in-depth information on genotypes and population composition of *Toxoplasma* from different regions of the country.

## Ethical considerations

Ethical issues (Including plagiarism, informed consent, misconduct, data fabrication and/or falsification, double publication and/or submission, redundancy, etc.) have been completely observed by the authors.
